# Quinazolinedione Derivatives as Potential Anticancer Agents Through Apoptosis Induction in MCF-7

**DOI:** 10.3390/ijms26136038

**Published:** 2025-06-24

**Authors:** Tanapol Limboonreung, Teetat Suansilpong, Panitan Jumjitvi, Duangporn Lohawittayanan, Sucheewin Krobthong, Sitthivut Charoensutthivarakul

**Affiliations:** 1 School of Dentistry, King Mongkut’s Institute of Technology Ladkrabang, Chalongkrung Road, Ladkrabang, Bangkok 10520, Thailand; 2 Innovative Molecular Discovery Laboratory (iMoD), School of Bioinnovation and Bio-Based Product Intelligence, Faculty of Science, Mahidol University, Bangkok 10400, Thailand; 3 Department of Chemistry, Faculty of Science, Chulalongkorn University, Bangkok 10330, Thailand; 4 Center for Neuroscience, Faculty of Science, Mahidol University, Bangkok 10400, Thailand

**Keywords:** programmed cell death, breast cancer, caspase activation, MCF-7, quinazolinedione derivatives

## Abstract

Breast cancer remains a leading cause of mortality among women worldwide. Surgery, radiation therapy, chemotherapy, and hormone-based treatments are standard therapeutic approaches, but drug resistance and adverse effects necessitate the search for novel anticancer agents. Quinazolinedione derivatives have emerged as potential anticancer compounds due to their cytotoxic and apoptosis-inducing properties. This study aimed to evaluate the apoptotic induction of previously reported quinazolinedione derivatives on MCF-7 breast cancer cells. The cytotoxic effect was assessed using the MTT assay, apoptosis was quantified by Annexin V-PE/7AAD staining and flow cytometry, and apoptosis-related protein expression was analyzed via multiplexed bead-based immunoassays. These findings indicate that two derivatives in the series significantly reduced the cell viability in a dose-dependent manner. Apoptosis was induced primarily through the intrinsic apoptotic pathway as evidenced by the upregulation of caspase-9 and p53 and the downregulation of Bcl-2 and p-Akt. These results highlight quinazolinedione derivatives as promising candidates for breast cancer therapy prompting further investigation into their molecular mechanisms and potential clinical applications.

## 1. Introduction

Breast cancer is the most frequently diagnosed malignancy among women and remains a major global health burden [1,2]. Although endocrine therapy using selective estrogen receptor modulators (SERMs), such as Fulvestrant, has improved treatment outcomes for estrogen receptor-positive (ER^+^) breast cancer, drug resistance and tumor recurrence remain significant challenges. Fulvestrant, in particular, is one of the most frequently prescribed drugs for hormone receptor-positive advanced breast carcinoma, owing to its ability to degrade the estrogen receptor and inhibit tumor proliferation [3].

Quinazolinedione derivatives represent a promising class of compounds with diverse biological activities including antimicrobial, anti-inflammatory, and antitumor properties, among others [4,5,6]. Structural modifications of quinazolinediones have led to the development of potent drugs, some of which have been successfully commercialized, saving millions of lives. For instance, quinazolinedione derivatives serve as key intermediates in the synthesis of antihypertensive drugs such as prazosin (Minipress^®^) and bunazosin (Detantol^®^) [7]. In recent years, several quinazolinedione-based compounds have emerged as potential anticancer agents. Notably, two derivatives bearing a 3-aminopyrrolidine moiety have shown cytotoxicity against MX-1 breast cancer cells through PARP-1/2 inhibition [8]. A previous report from our group demonstrated the potential of quinazolinedione derivatives as an antimalarial agent against *Plasmodium falciparum* (causative parasite of malaria) and as antitumor agent on MCF-7 breast cancer cells [9]. Amongst them, one derivative exhibited low cytotoxicity against MCF-7 cells; however, other structurally related derivatives from the series remain untested. Further exploration of these derivatives may uncover new antitumor candidates and contribute to the development of novel cancer therapies.

Apoptosis is a key target in cancer therapy, with chemotherapy being a well-established approach to induce this programmed cell death in cancer cells [10]. Two major apoptotic pathways including the extrinsic and intrinsic pathways are extensively studied in cancer therapy [11]. The extrinsic pathway is initiated by death receptors such as TNF (tumor necrosis factor) and the Fas ligand (Fas-L) [12]. These receptors trigger a cascade that activates caspase-8, leading to caspase-3 activation and apoptosis. The intrinsic pathway, primarily mitochondria-dependent, is triggered by cellular stressors like oxidative stress, radiation, and cytotoxic drugs [13]. The key event in the intrinsic pathway is the release of cytochrome c from mitochondria into the cytosol, which promotes apoptosome formation with Apaf-1 and caspase-9. Bcl-2, an anti-apoptotic protein, prevents cytochrome c release, an effect counteracted by the Bad protein. Caspase-9 activation subsequently leads to caspase-3 activation and apoptosis [14].

The balance between apoptosis and cell survival is tightly regulated by several key signaling proteins, including p53, JNK, and Akt. Among these, the tumor suppressor protein p53 plays a central role and is modulated through specific phosphorylation events. For instance, phosphorylation at Thr81, often induced by extrinsic apoptotic pathways via JNK activation, enhances p53 stability and pro-apoptotic function [15]. Additionally, p53 can be phosphorylated at Ser46 by the p38 kinase, leading to the activation of p53-responsive genes, conformational changes in the pro-apoptotic proteins Bax and Bak, release of cytochrome c from mitochondria, and subsequent induction of apoptosis [16]. Activated JNK, phosphorylated at Thr183/Tyr185, further promotes apoptosis by upregulating pro-apoptotic proteins and suppressing anti-apoptotic ones [17]. Interestingly, in a genetic model of breast cancer, JNK-deficient mice exhibited accelerated tumor progression despite the presence of p38 kinase, underscoring the critical role of JNK in tumor suppression [18]. Notably, the expression levels of p53, JNK, and p38 are generally reduced in breast cancer tissues, suggesting their potential roles in disease progression and evasion of apoptosis [19].

Akt, also known as protein kinase B, is a serine/threonine kinase that plays a critical role in regulating cell survival, growth, and proliferation—functions that are frequently dysregulated in cancer. Its activation is tightly controlled through a phosphorylation cascade. Specifically, Akt is phosphorylated at Ser473 by the mammalian target of rapamycin complex 2 (mTORC2), a key upstream regulator. Upon phosphorylation, Akt translocates from the plasma membrane into the cytoplasm and nucleus, where it modulates various downstream signaling pathways involved in cell survival and tumor progression [20]. One of the primary mechanisms by which Akt promotes cell survival is through its interaction with the Bcl-2 family of proteins [21,22]. Akt enhances the expression of the anti-apoptotic protein Bcl-2 and directly phosphorylates Bcl-2 at Ser70, thereby increasing its stability and reinforcing its anti-apoptotic function. This phosphorylation event at Ser70 is particularly significant, as it diminishes Bcl-2’s ability to bind to the pro-apoptotic protein Bad, ultimately tipping the balance in favor of cell survival by suppressing the intrinsic apoptotic pathway [23]. In addition to its effects on Bcl-2, Akt also exerts a suppressive influence on the tumor suppressor protein p53, which is a key mediator of apoptosis. Akt can indirectly lead to the ubiquitination and subsequent proteasomal degradation of p53, thereby preventing the activation of p53-dependent apoptotic genes [24]. Through these multifaceted mechanisms, Akt not only promotes resistance to apoptosis but also contributes to the aggressive and therapy-resistant nature of many cancers, including breast cancer.

Recent studies have reported the anticancer potential of several quinazolinedione derivatives, but their underlying mechanisms in breast cancer remain unclear. This study investigates the apoptotic effects of quinazolinedione derivatives on MCF-7 breast cancer cells by evaluating the cell viability, apoptosis induction ability, and alterations in the level of apoptosis-related protein expression to elucidate their potential mechanisms of action.

## 2. Results

### 2.1. Quinazolinedione Derivatives Inhibited MCF-7 Cell Viability

**Figure 1:**
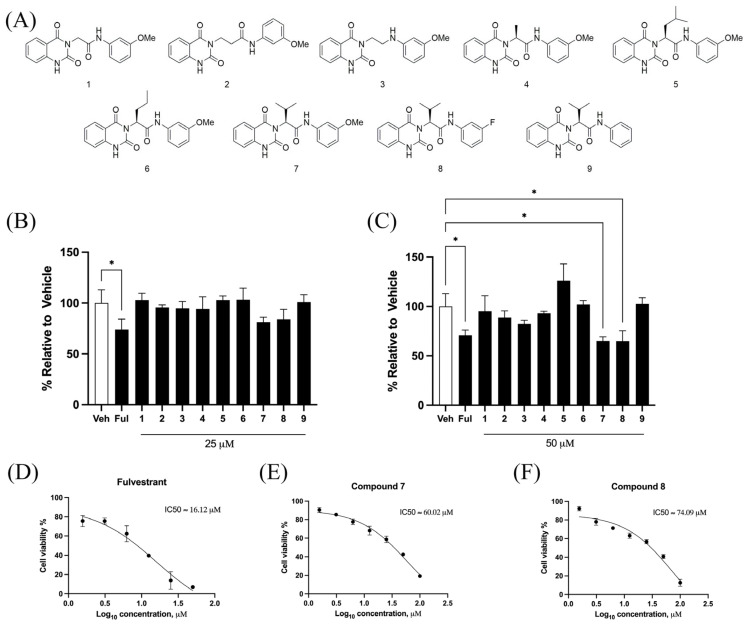
Effects of quinazolinedione derivatives on MCF-7 cell viability. (**A**) Chemical structures of the quinazolinedione derivatives (compound **1**–**9**) used in this study. (**B**) MCF-7 cells were treated with 25 µM of quinazolinedione derivatives for 72 h, and the cell viability was assessed using the MTT assay. (**C**) MCF-7 cells were treated with 50 µM of quinazolinedione derivatives for 72 h, showing a dose-dependent reduction in cell viability. (**D**–**F**) The IC_50_ value calculated by nonlinear regression analysis using a three-parameter Hill equation of Fulvestrant (**D**), compound **7** (**E**), and compound **8** (**F**) on MCF-7 cell viability over a range of concentrations. The results are expressed as % relative to the vehicle control (Veh). * *p* < 0.05 indicates statistical significance compared to vehicle control.

The effects of quinazolinedione derivatives on cell viability were evaluated in MCF-7 breast cancer cells using the MTT assay. The chemical structures of the tested quinazolinedione derivatives are presented in Figure 1A. Treatment with compound **7** and compound **8** at 50 μM for 72 h significantly reduced the cell viability compared to the 25 μM concentration (Figure 1B,C). Fulvestrant, a clinically approved breast cancer drug, was used as a positive control at 5 μM. Moreover, Fulvestrant, compound **7**, and compound **8** all demonstrated a dose-dependent reduction in MCF-7 cell viability (Figure 1D–F). The calculated IC_50_ values for Fulvestrant, compound **7**, and compound **8** were 16.12 μM, 60.02 μM, and 74.09 μM, respectively. Based on these results, compound **7** and compound **8** at 50 μM were selected for use in subsequent experiments.

### 2.2. Quinazolinedione Derivatives Induce Apoptosis in MCF-7 Cells

**Figure 2:**
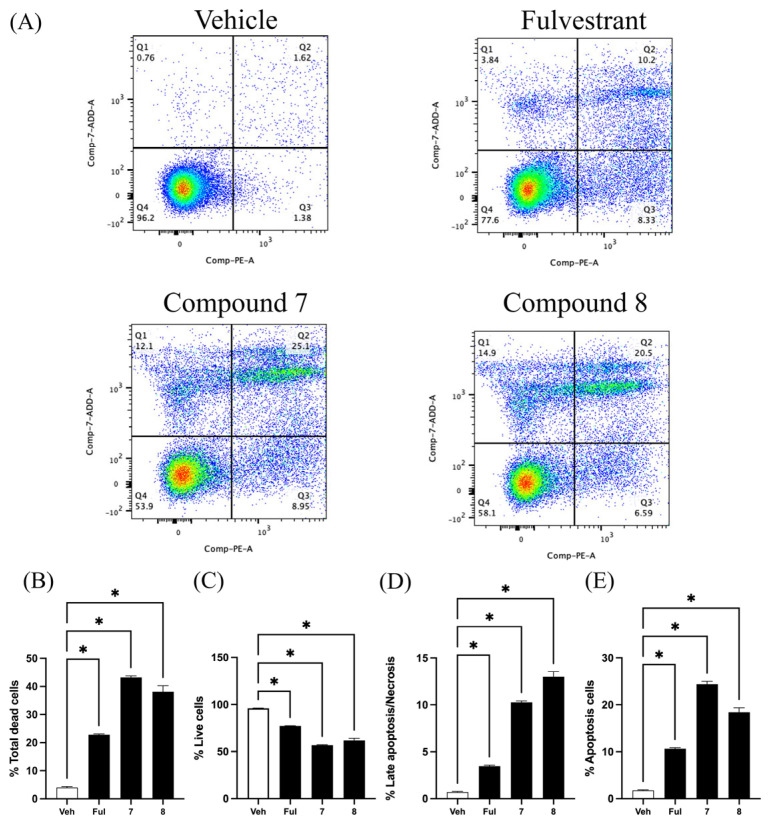
Quinazolinedione derivatives induce apoptosis in MCF-7 cells. (**A**) Representative flow cytometry dot plots of Annexin V-PE/7-AAD staining in MCF-7 cells treated with vehicle (Veh), Fulvestrant (Ful, 5 µM), compound **7** (50 µM), or compound **8** (50 µM) for 72 h. Quadrant analysis: Q1—necrotic cells (Annexin V^−^/7-AAD^+^), Q2—late apoptotic or necrotic cells (Annexin V^+^/7-AAD^+^), Q3—early apoptotic cells (Annexin V^+^/7-AAD^−^), and Q4—viable cells (Annexin V^−^/7-AAD^−^). (**B**) Quantification of total dead cells (sum of Q1, Q2, and Q3 populations). (**C**) Percentage of live cells (Q4). (**D**) Percentage of late apoptotic/necrotic cells (Q1). (**E**) Percentage of apoptotic cells (Q2). Data are presented as mean ± SEM. * *p* < 0.05 compared to vehicle (Veh); statistical comparisons were performed using one-way ANOVA followed by Tukey’s post hoc test.

Quinazolinedione derivatives promote phosphatidylserine externalization and apoptosis in MCF-7 cells. Apoptotic cell populations were evaluated using Annexin V-PE and 7-AAD double staining, followed by flow cytometry analysis. Cells positive for both Annexin V and 7-AAD (Q2) were classified as apoptotic, Annexin V-positive/7-AAD-negative cells (Q3) as early apoptotic, and 7-AAD-positive/Annexin V-negative cells (Q1) as late apoptotic or necrotic. Unstained cells (Q4) were considered viable (Figure 2A). Treatment with compound **7** and compound **8** at 50 μM significantly increased the total cell death in MCF-7 cells (Figure 2B), with total dead cell percentages of 43.2 ± 0.5% and 38.1 ± 2.1%, respectively. In comparison, treatment with 5 μM Fulvestrant resulted in 22.8 ± 0.2% total cell death. Specifically, compound **7** induced 24.4 ± 0.6% early apoptotic cells, and compound **8** induced 18.4 ± 0.9%, while Fulvestrant induced 10.6 ± 0.2%. These results suggest that both compound **7** and compound **8** induce apoptosis in MCF-7 cells effectively with compound **7** showing the strongest pro-apoptotic activity.

### 2.3. Quinazolinedione Derivatives Modulate Apoptosis-Related Protein Expression in MCF-7 Cells

**Figure 3:**
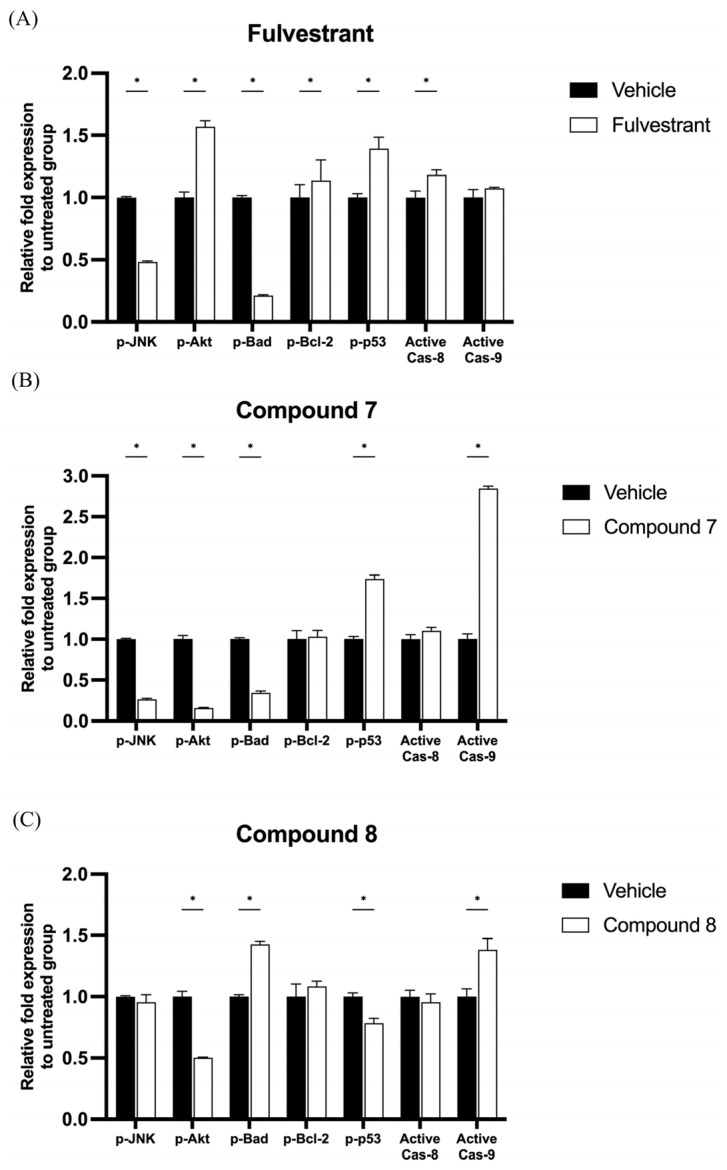
Quinazolinedione derivatives modulate apoptosis-related protein expression in MCF-7 cells. (**A**) Fulvestrant (5 µM), (**B**) compound **7** (50 µM), and (**C**) compound **8** (50 µM) treatments regulate key apoptosis-related protein expressions in MCF-7 cells compared to untreated controls. Prote in levels were measured using the MILLIPLEX^®^ Early Apoptosis 7-Plex Magnetic Bead Kit and detected via the Luminex^®^ system. p-JNK, p-Akt, p-Bad, p-Bcl-2, and p-p53 phosphorylation levels were assessed. Active caspase-8 and active caspase-9 were analyzed to determine apoptotic pathway activation. Statistical significance: * *p* < 0.05 compared to untreated controls.

To investigate the mechanism of quinazolinedione derivatives in MCF-7 breast cancer cells through the early apoptosis signaling pathway, the phosphorylated forms of JNK, Akt, Bad, Bcl-2, p53, active caspase-8, and active caspase-9 were captured using magnetic beads and detected by the Luminex^®^ system. In MCF-7 cells, Fulvestrant at a concentration of 5 μM significantly reduced the protein expression levels of p-JNK and p-Bad, while the expression levels of p-Akt, p-Bcl-2, p-p53, and active caspase-8 were significantly increased compared to the control group (Figure 3A). Moreover, compound **7** at a concentration of 50 μM significantly reduced the protein expression levels of p-JNK, p-Akt, and p-Bad, while increasing the expression levels of p-p53 and active caspase-9 compared to the control group (Figure 3B). Furthermore, compound **8** at a concentration of 50 μM significantly reduced the protein expression levels of p-Akt and p-p53, while increasing the expression levels of p-Bad and active caspase-9 compared to the control group (Figure 3C).

## 3. Discussion

Compound **7** and compound **8** are quinazolinedione derivatives prepared through lead optimization to enhance their antimalarial properties [4]. Various quinazolinedione derivatives have been developed for various applications, such as antimalarial, antimicrobial, and anticancer activities [5,6]. In this study, we found that both compound **7** and compound **8** exhibited significant anticancer effects against MCF-7 breast cancer cells in a dose-dependent manner, with compound **7** demonstrating slightly higher potency. These compounds not only reduced the MCF-7 cell viability but also increased the apoptotic cell populations. Mechanistically, compound **7** and compound **8** primarily induced apoptosis through caspase-9 activation, suggesting involvement of the intrinsic apoptotic pathway. 

In contrast, Fulvestrant, a clinically used anti-breast cancer drug, triggered apoptosis via caspase-8 activation, indicating engagement of the extrinsic pathway.

**Figure 4:**
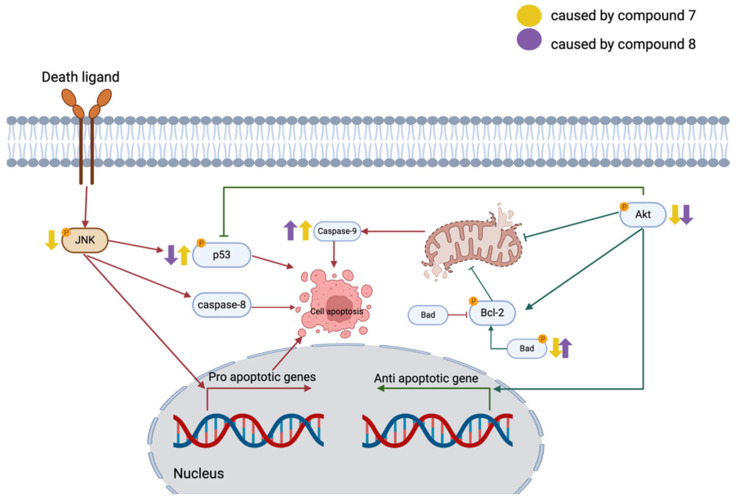
Proposed apoptotic signaling pathways modulated by quinazolinedione derivatives in MCF-7 cells. The molecular pathways affected by compound **7** (yellow arrows) and compound **8** (purple arrows) are based on protein expression profiles obtained from the MILLIPLEX^®^ Early Apoptosis 7-Plex Magnetic Bead Kit. Both compounds downregulate phosphorylated Akt (p-Akt), leading to reduced anti-apoptotic Bcl-2 activity and dephosphorylation of Bad, thereby promoting mitochondrial dysfunction and cytochrome c release. Activation of p53 and caspase-9 further supports intrinsic (mitochondrial) apoptosis. Compound **7** uniquely reduces phosphorylated JNK (p-JNK), while compound **8** increases p53 levels. Arrows indicate pro-apoptotic pathways (red) or anti-apoptotic pathways (green). (Created by BioRender.com/Mahidol University) (Version 1.0.0.4).

Breast cancer is one of the most devastating diseases worldwide. Estrogen receptor-positive (ER^+^) breast cancer utilizes circulating estrogen to drive tumor progression. As a result, estrogen antagonists have been widely used as therapeutic agents to inhibit estrogen signaling. Fulvestrant, a selective estrogen receptor degrader, is commonly prescribed for ER^+^ breast cancer. In the present study, Fulvestrant promoted apoptosis in MCF-7 breast cancer cells by activating caspase-8, a key initiator of the extrinsic apoptotic pathway. This finding is consistent with previous studies reporting Fulvestrant-induced apoptosis via caspase-8 activation [25]. Caspase-8 is known as a mitochondrial-independent apoptosis initiator [26]. Additionally, the present study suggests that Fulvestrant treatment increased the phosphorylation of Akt (Ser473) and p53 (Ser46) (Figure 4). Phosphorylation at Ser46 enhances p53 pro-apoptotic activity, reinforcing its role in programmed cell death [27]. However, the phosphorylation of Akt (Ser473) is typically associated with anti-apoptotic signaling and cell survival, which subsequently promotes Bcl-2 phosphorylation and contributes to drug resistance [28]. Interestingly, previous studies have suggested that in long-term low-dose anti-estrogen therapy, increased p-Akt (Ser473) levels may contribute to Fulvestrant resistance in breast cancer cells [29]. These findings highlight the complex interplay between pro-apoptotic and pro-survival signaling pathways in Fulvestrant-treated breast cancer cells. Further investigations into the molecular mechanisms underlying Akt-mediated resistance to Fulvestrant could provide valuable insights for improving therapeutic strategies, particularly for patients who develop resistance to endocrine therapy.

Compound **7** and compound **8** are two quinazolinedione derivatives that exhibit significant antitumor effects. The development of new anticancer drugs has increasingly focused on quinazolinedione-based compounds due to their structural versatility and ability to undergo modifications that enhance their pharmacological properties [30]. Previous studies have reported that various quinazolinedione derivatives possess promising antitumor activity, supporting their potential as chemotherapeutic agents [6]. In this study, the experiments screened a small library of quinazolinedione derivatives for their anticancer properties in MCF-7 breast cancer cells. Among them, compound **7** and compound **8** demonstrated the most potent cytotoxic effects, significantly reducing the MCF-7 cell viability. Further investigation revealed that both compounds induced apoptosis in MCF-7 cells, primarily through the activation of caspase-9 and a reduction in the phosphorylated Akt (Ser473) levels. Caspase-9 activation is a hallmark of mitochondria-dependent apoptosis, a pathway commonly exploited by chemotherapy drugs to induce cancer cell death [31]. Upon activation, caspase-9 initiates a cascade of apoptotic events, leading to the subsequent activation of executioner caspases, such as caspase-3 and caspase-7, ultimately resulting in programmed cell death. Unlike Fulvestrant, which increases phosphorylated Akt (Ser473) levels, compound **7** and compound **8** exert their anticancer effects by reducing Akt phosphorylation. This reduction suggests that these compounds may counteract Akt-mediated survival signaling, making them promising candidates for targeting breast cancer cells with dysregulated Akt signaling.

There are several strategies currently employed in the development of anti-breast cancer agents. Fulvestrant and tamoxifen, for example, act by targeting estrogen receptors and disrupting estrogen-mediated signaling, ultimately inducing apoptosis [32]. Non-specific cancer cell type chemotherapeutic agents such as doxorubicin and 5-fluorouracil (5-FU) are also commonly used in breast cancer treatment. Doxorubicin functions by inhibiting topoisomerase II, leading to DNA damage, while 5-FU inhibits thymidylate synthase, an enzyme critical for DNA synthesis [33,34]. Despite their efficacy, chemoresistance remains a persistent clinical challenge [35]. To address this, new therapeutic strategies have emerged. LY01005, a GnRH agonist currently in clinical trials, aims to reduce estrogen levels [36], while Everolimus, an mTOR inhibitor, exerts anti-breast cancer effects through inhibition of the PI3K/Akt/mTOR pathway and activation of caspase-8-mediated apoptosis [37,38]. In this study, we observed that Fulvestrant primarily activated caspase-8, while compounds **7** and **8** induced apoptosis via caspase-9 activation, suggesting a distinct mechanism involving the intrinsic mitochondrial pathway. This mechanistic diversity may be advantageous in combination therapies and warrants further investigation into the broader molecular targets, specificity, and potential clinical applications of these novel quinazolinedione derivatives [39].

## 4. Materials and Methods

### 4.1. Quinazolinedione Derivatives

A library of quinazolinedione derivatives (compounds **1**–**9**) was synthesized following the published protocol, and the compound characterizations were described previously [4]. The compounds were dissolved in dimethyl sulfoxide (DMSO, Sigma, St. Louis, MO, USA) to achieve 10 mM stock solutions and were stored under −20 °C until use.

### 4.2. Cell Culture and Maintenance

The human breast cancer cell line MCF-7 was purchased from The American Type Culture Collection (ATCC: HTB-22™, Manassas, VA, USA). The cells were cultured using Dulbecco’s Modified Eagle Medium (DMEM, Gibco, Thermo Fisher Scientific, Waltham, MA, USA), supplemented with 10% Fetal Bovine Serum (FBS) and 1% Penicillin-Streptomycin (Gibco), and were maintained in a CO_2_ incubator at 37 °C and 95% humidity (nu-4750, Nuaire, Plymouth, MN, USA).

The subculture was achieved by trypsinization. The cells were washed twice by 37 °C PBS (Phosphate Buffered saline, pH 7.4, Gibco). Then, trypsin-EDTA (0.25%, Gibco) was added and incubated for 4 min at 37 °C. The cells were resuspended in cultured media and counted using a hemocytometer (Thermo, Waltham, MA, USA). The desired cell number was calculated and transferred into a new vessel.

### 4.3. Cell Viability Test (MTT Test)

MCF-7 cells were plated onto 96-well plates at 8000 cells/well density. The cells were left to adhere for 24 h. The plates were then incubated with different concentrations of quinazolinedione derivatives, 5 μM Fulvestrant (Tocris Bioscience, Avonmouth, UK) as positive control, and 0.5% DMSO as a vehicle. Following the incubation period, the cells were incubated with MTT (3-(4,5-dimethylthiazolyl-2)-2,5-diphenyltetrazolium bromide) (Sigma-Aldrich, Burlington, MA, USA) at 0.5 mg/mL for 4 h. The formazan crystal was solubilized in DMSO and analyzed spectrophotometrically (570 nm) by Agilent BioTek Cytation 7 (Biotek, Winooski, VT, USA). The results were expressed as the relative viability compared to the vehicle group.

To determine the IC_50_ values of the compounds, MCF-7 cells were treated with a range of concentrations (0–100 μM) of each compound for 72 h. All treatment and control groups contained 1% DMSO as the final vehicle concentration. The cell viability was assessed using the MTT assay. The percentage of viable cells was plotted against the log_10_-transformed concentrations, and the IC_50_ values were calculated by nonlinear regression analysis using a three-parameter Hill equation (log[inhibitor] vs. normalized response–variable slope) in GraphPad Prism 8.

### 4.4. Apoptosis Detection Assay

Apoptosis detection was performed using the Annexin V-PE Early Apoptosis Detection Kit (Cell Signaling, Danvers, MA, USA, #40295), following the manufacturer’s instructions. After incubation, 1 × 10^5^ cells were trypsinized and resuspended in 100 µL of binding buffer. Then, 2 µL of Annexin V-Phycoerythrin (PE) conjugate and 10 µL of 7-Aminoactinomycin D (7AAD) were added to the suspension and incubated for 10 min. The samples were then diluted in 250 µL of binding buffer and immediately analyzed using a BD FACSCanto™ flow cytometer (BD biosciences, Franklin Lakes, NJ, USA). Cells with double labeling were classified as being in the late apoptosis stage and were expressed as a percentage of the late apoptotic cells relative to the total cell population.

### 4.5. Early Apoptosis Protein Detection

Protein expression related to apoptosis was analyzed using the MILLIPLEX^®^ Early Apoptosis 7-Plex Magnetic Bead Kit (Merck Millipore, Burlington, MA, USA). The assay detected seven apoptosis-related proteins, including phosphorylation of Akt (S473), Bad (Ser112), Bcl-2 (Ser70), p53 (Ser46), JNK (Thr183/Tyr185), active Caspase-8 (Asp384), and active Caspase-9 (Asp315). Briefly, cells were lysed with ice-cold MILLIPLEX^®^ Lysis Buffer containing a Protease Inhibitor Cocktail (Sigma-Aldrich). The cells were then scraped off and incubated for 10 min at 4 °C. The lysates were centrifuged at 12,000× *g* for 10 min at 4 °C, and the protein concentration was quantified using the BCA assay (Pierce™, Thermo Scientific, Waltham, MA, USA). The lysates were then mixed with magnetic beads and detected using MILLIPLEX^®^ Streptavidin–Phycoerythrin antibodies with the Luminex^®^ xMAP INTELLIFLEX DR-SE System. GAPDH MAPmate™ (Merck Millipore, Burlington, MA, USA) was used as an internal control. Data were expressed as the relative expression levels normalized to the control.

### 4.6. Statistical Analysis

All experiments were independently performed at least three times (*n* = 3) and expressed as the mean values. Statistical significance was determined using one-way ANOVA with Tukey’s post hoc *t*-test. Any group with a *p*-value of less than 0.05 was considered statistically significant.

## 5. Conclusions

Quinazolinedione derivatives 7 and 8 exhibit potent apoptotic effects in MCF-7 breast cancer cells, primarily through caspase-9 activation and downregulation of phosphorylated Akt (Ser473). These findings suggest that these quinazolinedione derivatives may serve as promising anticancer agents. However, further studies are required to evaluate their in vivo efficacy and to explore potential synergistic effects with existing therapies.

## Data Availability

All data supporting the results are available within the article.

## References

[1] Giaquinto A.N., Sung H., Newman L.A., Freedman R.A., Smith R.A., Star J., Jemal A., Siegel R.L. (2024). Breast Cancer Statistics 2024. CA A Cancer J. Clin..

[2] Kinnel B., Singh S.K., Oprea-Ilies G., Singh R. (2023). Targeted Therapy and Mechanisms of Drug Resistance in Breast Cancer. Cancers.

[3] Osborne C.K., Wakeling A., Nicholson R.I. (2004). Fulvestrant: An Oestrogen Receptor Antagonist with a Novel Mechanism of Action. Br. J. Cancer.

[4] Charoensutthivarakul S., Lohawittayanan D., Kanjanasirirat P., Jearawuttanakul K., Seemakhan S., Chabang N., Schlaeppi P., Tantivess V., Limboonreung T., Phanchana M. (2023). Rational Design and Lead Optimisation of Potent Antimalarial Quinazolinediones and Their Cytotoxicity against MCF-7. Molecules.

[5] Almela M.J., Lozano S., Lelièvre J., Colmenarejo G., Coterón J.M., Rodrigues J., Gonzalez C., Herreros E. (2015). A New Set of Chemical Starting Points with Plasmodium Falciparum Transmission-Blocking Potential for Antimalarial Drug Discovery. PLoS ONE.

[6] Gouhar R.S., Kamel M.M. (2018). Synthesis and Reactions of Some New Quinazoline Derivatives for In Vitro Evaluation as Anticancer and Antimicrobial Agents. J. Heterocycl. Chem..

[7] Patil Y.P., Tambade P.J., Jagtap S.R., Bhanage B.M. (2008). Cesium Carbonate Catalyzed Efficient Synthesis of Quinazoline-2,4(1H,3H)-Diones Using Carbon Dioxide and 2-Aminobenzonitriles. Green Chem. Lett. Rev..

[8] Zhou J., Ji M., Yao H., Cao R., Zhao H., Wang X., Chen X., Xu B. (2018). Discovery of Quinazoline-2,4(1H,3H)-Dione Derivatives as Novel PARP-1/2 Inhibitors: Design, Synthesis and Their Antitumor Activity. Org. Biomol. Chem..

[9] Charoensutthivarakul S., Lohawittayanan D., Kanjanasirirat P., Jearawuttanakul K., Seemakhan S., Borwornpinyo S., Phanchana M. (2022). A Concise Synthesis Towards Antimalarial Quinazolinedione TCMDC-125133 and Its Anti-Proliferative Activity against MCF-7. Molbank.

[10] Ricci M.S., Zong W.-X. (2006). Chemotherapeutic Approaches for Targeting Cell Death Pathways. Oncologist.

[11] Jan R., Chaudhry G.-S. (2019). Understanding Apoptosis and Apoptotic Pathways Targeted Cancer Therapeutics. Adv. Pharm. Bull..

[12] Mongkolsapaya J., Grimes J.M., Chen N., Xu X.N., Stuart D.I., Jones E.Y., Screaton G.R. (1999). Structure of the TRAIL-DR5 Complex Reveals Mechanisms Conferring Specificity in Apoptotic Initiation. Nat. Struct. Biol..

[13] Ghavami S., Kerkhoff C., Los M., Hashemi M., Sorg C., Karami-Tehrani F. (2004). Mechanism of Apoptosis Induced by S100A8/A9 in Colon Cancer Cell Lines: The Role of ROS and the Effect of Metal Ions. J. Leukoc. Biol..

[14] Yuan S., Akey C.W. (2013). Apoptosome Structure, Assembly, and Procaspase Activation. Structure.

[15] Shi Y., Nikulenkov F., Zawacka-Pankau J., Li H., Gabdoulline R., Xu J., Eriksson S., Hedström E., Issaeva N., Kel A. (2014). ROS-Dependent Activation of JNK Converts P53 into an Efficient Inhibitor of Oncogenes Leading to Robust Apoptosis. Cell Death Differ..

[16] Perfettini J.-L., Castedo M., Nardacci R., Ciccosanti F., Boya P., Roumier T., Larochette N., Piacentini M., Kroemer G. (2005). Essential Role of P53 Phosphorylation by P38 MAPK in Apoptosis Induction by the HIV-1 Envelope. J. Exp. Med..

[17] Dhanasekaran D.N., Reddy E.P. (2008). JNK Signaling in Apoptosis. Oncogene.

[18] Itah Z., Chaudhry S., Raju Ponny S., Aydemir O., Lee A., Cavanagh-Kyros J., Tournier C., Muller W.J., Davis R.J. (2023). HER2-Driven Breast Cancer Suppression by the JNK Signaling Pathway. Proc. Natl. Acad. Sci. USA.

[19] Yeh Y.-T., Hou M.-F., Chung Y.-F., Chen Y.-J., Yang S.-F., Chen D.-C., Su J.-H., Yuan S.-S.F. (2006). Decreased Expression of Phosphorylated JNK in Breast Infiltrating Ductal Carcinoma Is Associated with a Better Overall Survival. Int. J. Cancer.

[20] Hresko R.C., Mueckler M. (2005). mTOR.RICTOR Is the Ser473 Kinase for Akt/Protein Kinase B in 3T3-L1 Adipocytes. J. Biol. Chem..

[21] Kondo E., Miyake T., Shibata M., Kimura T., Iwagaki H., Nakamura S.-I., Tanaka T., Ohara N., Ichimura K., Oka T. (2005). Expression of Phosphorylated Ser70 of Bcl-2 Correlates with Malignancy in Human Colorectal Neoplasms. Clin. Cancer Res..

[22] Low I.C.C., Loh T., Huang Y., Virshup D.M., Pervaiz S. (2014). Ser70 Phosphorylation of Bcl-2 by Selective Tyrosine Nitration of PP2A-B56δ Stabilizes Its Antiapoptotic Activity. Blood.

[23] Ruvolo P.P., Deng X., May W.S. (2001). Phosphorylation of Bcl2 and Regulation of Apoptosis. Leukemia.

[24] Chibaya L., Karim B., Zhang H., Jones S.N. (2021). Mdm2 Phosphorylation by Akt Regulates the P53 Response to Oxidative Stress to Promote Cell Proliferation and Tumorigenesis. Proc. Natl. Acad. Sci. USA.

[25] Dolfi S.C., Jäger A.V., Medina D.J., Haffty B.G., Yang J.-M., Hirshfield K.M. (2014). Fulvestrant Treatment Alters MDM2 Protein Turnover and Sensitivity of Human Breast Carcinoma Cells to Chemotherapeutic Drugs. Cancer Lett..

[26] Zhong B., Liu M., Bai C., Ruan Y., Wang Y., Qiu L., Hong Y., Wang X., Li L., Li B. (2020). Caspase-8 Induces Lysosome-Associated Cell Death in Cancer Cells. Mol. Ther..

[27] Feng L., Hollstein M., Xu Y. (2006). Ser46 Phosphorylation Regulates P53-Dependent Apoptosis and Replicative Senescence. Cell Cycle.

[28] Zhou H., Li X.-M., Meinkoth J., Pittman R.N. (2000). Akt Regulates Cell Survival and Apoptosis at a Postmitochondrial Level. J. Cell Biol..

[29] Fu X., Creighton C.J., Biswal N.C., Kumar V., Shea M., Herrera S., Contreras A., Gutierrez C., Wang T., Nanda S. (2014). Overcoming Endocrine Resistance Due to Reduced PTEN Levels in Estrogen Receptor-Positive Breast Cancer by Co-Targeting Mammalian Target of Rapamycin, Protein Kinase B, or Mitogen-Activated Protein Kinase Kinase. Breast Cancer Res..

[30] Gheidari D., Mehrdad M., Maleki S. (2022). The Quinazoline-2,4(1H,3H)-Diones Skeleton: A Key Intermediate in Drug Synthesis. Sustain. Chem. Pharm..

[31] Schwartz P.S., Waxman D.J. (2001). Cyclophosphamide Induces Caspase 9-Dependent Apoptosis in 9L Tumor Cells. Mol. Pharmacol..

[32] Shoda T., Kato M., Fujisato T., Demizu Y., Inoue H., Naito M., Kurihara M. (2017). Tamoxifen and Fulvestrant Hybrids Showed Potency as Selective Estrogen Receptor Down-Regulators. Med. Chem..

[33] Sinha B.K., Mimnaugh E.G., Rajagopalan S., Myers C.E. (1989). Adriamycin Activation and Oxygen Free Radical Formation in Human Breast Tumor Cells: Protective Role of Glutathione Peroxidase in Adriamycin Resistance. Cancer Res..

[34] Cameron D.A., Gabra H., Leonard R.C. (1994). Continuous 5-Fluorouracil in the Treatment of Breast Cancer. Br. J. Cancer.

[35] Cao J., Zhang M., Wang B., Zhang L., Zhou F., Fang M. (2021). Chemoresistance and Metastasis in Breast Cancer Molecular Mechanisms and Novel Clinical Strategies. Front. Oncol..

[36] Shao X.Y., Zhang Q.Y., Niu Z.F., Li M., Wang J.F., Chen Z.Z., Luo R.Z., Qiao G.D., Wang J.G., Qian L.Y. (2025). [Phase Ⅲ, multicenter, randomized comparative study of LY01005 and Zoladex^®^ for patients with premenopausal breast cancer]. Zhonghua Zhong Liu Za Zhi.

[37] Baselga J., Campone M., Piccart M., Burris H.A., Rugo H.S., Sahmoud T., Noguchi S., Gnant M., Pritchard K.I., Lebrun F. (2012). Everolimus in Postmenopausal Hormone-Receptor-Positive Advanced Breast Cancer. N. Engl. J. Med..

[38] Du L., Li X., Zhen L., Chen W., Mu L., Zhang Y., Song A. (2018). Everolimus Inhibits Breast Cancer Cell Growth through PI3K/AKT/mTOR Signaling Pathway. Mol. Med. Rep..

[39] Sammons S., Kornblum N.S., Blackwell K.L. (2019). Fulvestrant-Based Combination Therapy for Second-Line Treatment of Hormone Receptor-Positive Advanced Breast Cancer. Target. Oncol..

